# LKB1 and AMPK and the cancer-metabolism link - ten years after

**DOI:** 10.1186/1741-7007-11-36

**Published:** 2013-04-15

**Authors:** D Grahame Hardie, Dario R Alessi

**Affiliations:** 1 Division of Cell Signalling and Immunology, College of Life Sciences, University of Dundee, Dundee, DD1 5EH, Scotland, UK; 2 MRC Protein Phosphorylation Unit, College of Life Sciences, University of Dundee, Dundee, DD1 5EH, Scotland, UK

## Abstract

The identification of a complex containing the tumor suppressor LKB1 as the critical upstream kinase required for the activation of AMP-activated protein kinase (AMPK) by metabolic stress was reported in an article in *Journal of Biology *in 2003. This finding represented the first clear link between AMPK and cancer. Here we briefly discuss how this discovery came about, and describe some of the insights, especially into the role of AMPK in cancer, that have followed from it.

In September 2003, our groups published a joint paper [1] in *Journal of Biology *(now *BMC Biology*) that identified the long-sought and elusive upstream kinase acting on AMP-activated protein kinase (AMPK) as a complex containing LKB1, a known tumor suppressor. Similar findings were reported at about the same time by David Carling and Marian Carlson [2] and by Reuben Shaw and Lew Cantley [3]; at the time of writing these three papers have received between them a total of over 2,000 citations. These findings provided a direct link between a protein kinase, AMPK, which at the time was mainly associated with regulation of metabolism, and another protein kinase, LKB1, which was known from genetic studies to be a tumor suppressor. While the idea that cancer is in part a metabolic disorder (first suggested by Warburg in the 1920s [4]) is well recognized today [5], this was not the case in 2003, and our paper perhaps contributed towards its renaissance. The aim of this short review is to recall how we made the original finding, and to discuss some of the directions that these findings have taken the field in the ensuing ten years.

## AMPK as an energy sensor and metabolic switch

**Figure 1:**
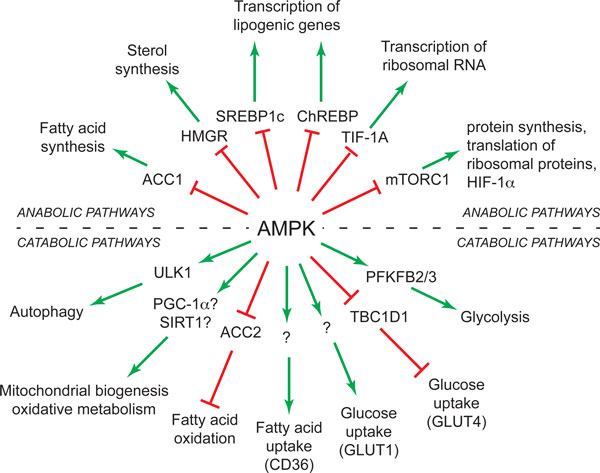
**Summary of a selection of target proteins and metabolic pathways regulated by AMPK**. Anabolic pathways switched off by AMPK are shown in the top half of the 'wheel' and catabolic pathways switched on by AMPK in the bottom half. Where a protein target for AMPK responsible for the effect is known, it is shown in the inner wheel; a question mark indicates that it is not yet certain that the protein is directly phosphorylated. For original references see [54]. Key to acronyms: ACC1/ACC2, acetyl-CoA carboxylases-1/-2; HMGR, HMG-CoA reductase; SREBP1c, sterol response element binding protein-1c; CHREBP, carbohydrate response element binding protein; TIF-1A, transcription initiation factor-1A; mTORC1, mechanistic target-of-rapamycin complex-1; PFKFB2/3, 6-phosphofructo-2-kinase, cardiac and inducible isoforms; TBC1D1, TBC1 domain protein-1; SIRT1, sirtuin-1; PGC-1α, PPAR-γ coactivator-1α; ULK1, Unc51-like kinase-1.

AMPK was discovered as a protein kinase activity that phosphorylated and inactivated two key enzymes of fatty acid and sterol biosynthesis: acetyl-CoA carboxylase (ACC) and 3-hydroxy-3-methylglutaryl-CoA reductase (HMGR). The ACC kinase activity was reported to be activated by 5'-AMP [6], and the HMGR kinase activity by reversible phosphorylation [7], but for many years the two activities were thought to be due to distinct enzymes. However, in 1987 the DGH laboratory showed that both were functions of a single protein kinase [8], which we renamed AMPK after its allosteric activator, 5'-AMP [9]. It was subsequently found that AMPK regulated not only lipid biosynthesis, but also many other metabolic pathways, both by direct phosphorylation of metabolic enzymes, and through longer-term effects mediated by phosphorylation of transcription factors and co-activators. In general, AMPK switches off anabolic pathways that consume ATP and NADPH, while switching on catabolic pathways that generate ATP (Figure 1). Findings that AMPK is activated in skeletal muscle during exercise [10] and that it increases muscle glucose uptake and fatty acid oxidation [11] led to the suggestion that AMPK-activating drugs might be useful for treating type 2 diabetes [12]. Indeed, it turned out that AMPK is activated by metformin, a drug that had at that time been used to treat type 2 diabetes for over 40 years, [13], and by phenformin [1], a closely related drug that had been withdrawn for treatment of diabetes due to side effects of lactic acidosis.

Metformin and phenformin are biguanides that inhibit mitochondrial function and so deplete ATP by inhibiting its production [14]. AMPK is activated by any metabolic stress that depletes ATP, either by inhibiting its production (as do hypoxia, glucose deprivation, and treatment with biguanides) or by accelerating its consumption (as does muscle contraction [10]). By switching off anabolism and other ATP-consuming processes and switching on alternative ATP-producing catabolic pathways, AMPK acts to restore cellular energy homeostasis. When ATP is consumed, both ADP and AMP increase, and it has recently become clear that both of the latter are activators of AMPK. ATP competes with AMP and ADP for binding to regulatory sites on the enzyme, thus preventing activation; activation of AMPK therefore depends on the ratios of ATP:ADP and AMP:ATP, and in this way the enzyme acts as a sensor of the metabolic state of the cell.

**Figure 2:**
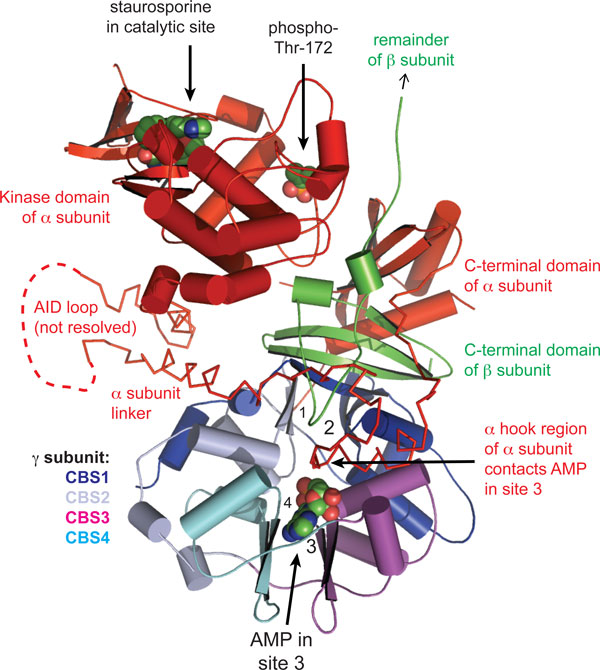
**Structure graphic of AMPK**. The α subunit is in red, the β subunit in green, and the four CBS motifs of the γ subunit in various shades of blue, magenta and cyan. For this structure, an AMPK complex phosphorylated on Thr172, lacking most of the β subunit and also a flexible loop from the α subunit, was crystallized in the presence of AMP and the kinase inhibitor staurosporine. For clarity, only the AMP in site 3 is shown, but the approximate location of binding clefts 1, 2 and 4 are also shown; in this view, sites 1 and 4 are at the back of the γ subunit and sites 2 and 3 at the front. The carboxy-terminal domain of the β subunit forms the core of the complex, bridging the carboxy-terminal domain of the α subunit and the γ subunit. Note the extended α subunit linker peptide between the kinase domain and carboxy-terminal domain, with its 'α hook' region contacting AMP in site 3. AID: α subunit autoinhibitory domain (this domain is believed to hold the catalytic domain in an inactive conformation in the absence of the regulatory domains). Drawn using MacPyMOL with Protein Database Entry 2Y94 [15].

AMPK has two regulatory subunits, β and γ, along with its catalytic α subunit (Figure 2). Like most kinases, it is activated by phosphorylation of a residue within the activation loop of the kinase domain (Thr172). The γ subunit contains four tandem sequence repeats known as CBS repeats, which are arranged in a pseudo-symmetrical manner to yield four potential adenine nucleotide-binding clefts. One of these (site 2) appears always to be unoccupied, one (site 4) appears to have permanently bound AMP, while the remaining two (1 and 3) bind AMP, ADP or ATP in competition [15,16]. In an unstressed cell where ATP:ADP ratios are high, sites 1 and 3 are probably largely occupied by ATP, but when a cell undergoes metabolic stress the concentrations of ADP and AMP increase relative to ATP, and they will progressively replace ATP at sites 1 and 3. Thr172 appears to be phosphorylated constantly by the upstream kinase, but under unstressed conditions it is immediately dephosphorylated, so that the net phosphorylation state remains close to zero. However, binding of ADP and/or AMP to the γ subunit both promotes phosphorylation and inhibits dephosphorylation of Thr172, causing a sensitive switch to the active, phosphorylated form. In addition, binding of AMP (but not ADP) causes a further allosteric activation of the phosphorylated kinase; this additional mechanism may further amplify the response in a severely stressed cell in which AMP levels are high.

This leaves open the question of the activating kinase that phosphorylates Thr172. It had been known since studies of 'HMGR kinase' in 1978 [7] that AMPK was activated by a distinct upstream kinase, but its identity had remained enigmatic.

## Discovery of the link between LKB1 and AMPK

Although by 1996 the DGH laboratory had partially purified an upstream kinase from rat liver that phosphorylated Thr172 [17], we had been unable to identify it. The breakthrough came when we switched to the yeast *Saccharomyces cerevisiae*, which contains a protein kinase (the SNF1 complex) that is a clear orthologue of AMPK [18,19]. The *S. cerevisiae *genome sequence had just been completed, and we screened a yeast kinome expression library for kinases that activated mammalian AMPK. This yielded Elm1 as a single hit, although, frustratingly, knocking out the *ELM1 *gene did not produce the same phenotype as knocking out *SNF1*. However, Martin Schmidt's group had just shown that a closely related kinase, Pak1 (now called Sak1) could also phosphorylate and activate the SNF1 complex [20], and working with him we observed the expected phenotype by making a triple knockout not only of Elm1 and Sak1 but also of Tos3, a third closely related kinase [21]. The Carling and Carlson groups simultaneously identified the same three upstream kinases [22].

**Figure 3:**
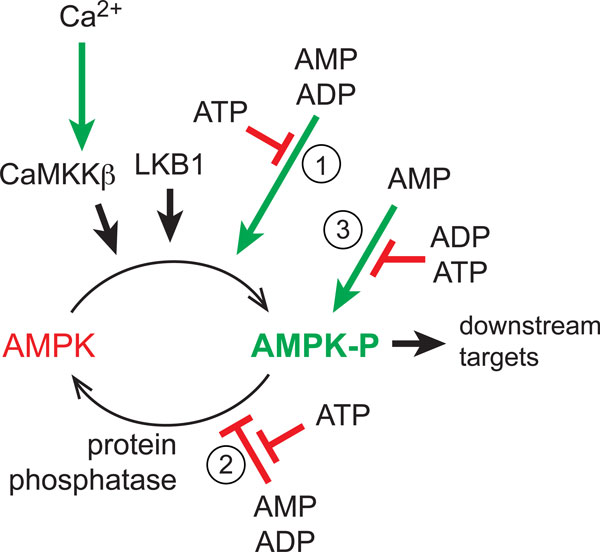
**Regulation of AMPK**. AMPK can be activated by increases in cellular AMP:ATP or ADP:ATP ratio, or Ca^2+^ concentration. AMPK is activated >100-fold on conversion from a dephosphorylated form (AMPK) to a form phosphorylated at Thr172 (AMPK-P) catalyzed by at least two upstream kinases: LKB1, which appears to be constitutively active, and CaMKKβ, which is only active when intracellular Ca^2+^ increases. Increases in AMP or ADP activate AMPK by three mechanisms: (1) binding of AMP or ADP to AMPK, causing a conformational change that promotes phosphorylation by upstream kinases (usually this will be LKB1, unless [Ca^2+^] is elevated); (2) binding of AMP or ADP, causing a conformational change that inhibits dephosphorylation by protein phosphatases; (3) binding of AMP (and not ADP), causing allosteric activation of AMPK-P. All three effects are antagonized by ATP, allowing AMPK to act as an energy sensor.

Although there were no clear orthologues of Elm1, Sak1 or Tos3 in mammals, the human kinase with closest sequence similarity (at least within the kinase domain) was LKB1 (liver kinase B1), which had been identified five years earlier as a tumor suppressor mutated in an inherited cancer susceptibility called Peutz-Jeghers syndrome [23,24]. The DRA laboratory had started to study LKB1 at that time, and had made the key observation that the enzyme was only active as a complex with two accessory subunits called STRAD and MO25 [25,26]. Working together, we were quickly able to demonstrate (using antibodies that DRA had generated) that the upstream kinase that the DGH group had been trying to purify from rat liver was indeed an LKB1-STRAD-MO25 complex. We also showed that activation of AMPK by metabolic stress in LKB1-null cells was defective, but could be rescued by re-expression of LKB1. These findings formed the basis of our paper in *Journal of Biology *[1]. The LKB1 complex was not itself regulated by AMP, with the effects of AMP or ADP on Thr172 phosphorylation being due instead, as described above, to binding of the nucleotides to the substrate, AMPK, causing conformational changes that promote phosphorylation and inhibit dephosphorylation [15,27-29] (Figure 3).

## The immediate aftermath (1): more kinases downstream of LKB1

**Figure 4:**
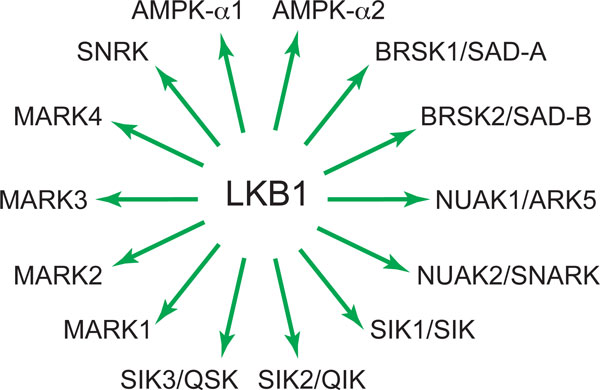
**Members of the AMPK and AMPK-related kinase (ARK) family**. All the kinases named in the figure are phosphorylated and activated by LKB1, although what regulates this phosphorylation is known only for AMPK. Alternative names are shown, where applicable.

While it was clear at that time that most kinases require phosphorylation in the activation loop to become active, it was also self-evident that there could not be a specific upstream kinase for every downstream kinase. The DRA group had previously identified PDK1 as the upstream kinase for PKB/Akt [30], and shown that it was also upstream of several other members of the AGC kinase family, introducing the concept of 'master upstream kinases' [31]. The two catalytic subunit isoforms of AMPK (α1 and α2) lie on a small sub-branch of the CaMK kinase family [32] that also contains 12 other kinases now referred to as the AMPK-related kinases or ARKs. Since the sequence around the site equivalent to Thr172 is highly conserved between AMPK and the ARKs, it seemed possible that LKB1 was a master kinase upstream of the whole subfamily, and this was soon shown to be the case [33,34] (Figure 4). Indeed, LKB1 activates all 12 ARKs by phosphorylating the residue equivalent to Thr172, and their activity is greatly diminished in LKB1-deficient cells.

Although the roles of the ARKs are poorly understood by comparison with those of AMPK, initial studies suggest that they play critical roles in regulating aspects of cell polarity (MARK, BRSK/SAD), cell proliferation (NUAKs) and CREB-regulated gene transcription (SIKs). The catalytic subunits of the ARKs do not interact with the γ subunits that provide the AMP/ADP sensing function of the AMPK complex [35], so they are not regulated by energy stress like AMPK, and indeed it is not yet understood how their activity and phosphorylation is regulated at the molecular level. As discussed below, however, their existence complicates the interpretation of the metabolic and tumor suppressor effect of LKB1, in particular in the case of the NUAKs, which appear to be involved in regulation of cell proliferation, and the SIKs, involved in the regulation of gluconeogenesis.

## The immediate aftermath (2): more kinases upstream of AMPK

Our 2003 paper [1] showed that although genetic loss of LKB1 in mammalian cells greatly reduces phosphorylation of AMPK on Thr172, it does not eliminate it entirely, suggesting that other kinases must also phosphorylate the site. The DGH group had reported in 1995 that calmodulin-dependent kinase kinases (CaMKKs) could activate AMPK [27], although it was not clear at the time that this was physiologically relevant. However, DGH and others soon reported that CaMKKs (especially the CaMKKβ isoform) did indeed provide an alternative Ca^2+^-stimulated pathway by which AMPK could be activated in intact cells in the absence of LKB1 [36-38]. This pathway accounts for AMPK activation in neurones exposed to membrane depolarization [36], in T cells activated via the antigen receptor [39], and in cells treated with ligands for G protein-coupled receptors that trigger intracellular release of inositol trisphosphate and hence Ca^2+^. The latter include endothelial cells treated with thrombin [40], and hypothalamic neurones involved in appetite control treated with the 'hunger hormone' ghrelin [41,42]. Interestingly, despite the very close sequence similarities within the activation loops of AMPK and the ARKs, none of the ARKs appear to be phosphorylated and activated by CaMKKβ [43].

## New findings arising from genetic ablation of LKB1

Null mutations of either LKB1 or AMPK are embryonic lethal in mice, which means that conditional knockouts are necessary to study their function *in vivo*. This is, however, complicated in the case of AMPK by the existence of two or three functionally redundant isoforms of each of the three subunits, so that genes encoding all isoforms of one subunit must be knocked out to produce a phenotype. The identification of LKB1 as the upstream kinase activating AMPK simplified the investigation of AMPK function *in vivo*, because LKB1 is encoded by a single gene. It allowed the demonstration, for example, that mice with a muscle-specific knockout of LKB1, in which AMPK was no longer activated by contraction, failed to display the normal increase in muscle glucose uptake in response to contraction [44]. The convenience of having to knock out only a single gene is counterbalanced, however, by the status of LKB1 as a master upstream kinase that also acts upstream of the ARKs: thus, a phenotype caused by a tissue-specific LKB1 knockout cannot simply be ascribed to loss of AMPK activation without additional evidence. In the case of the muscle-specific LKB1 knockout, such evidence has been provided by a later study showing that skeletal muscle in which both AMPK-β subunits are knocked out to ablate AMPK activity have a rather similar phenotype [45]. By contrast, distinct effects of knocking out LKB1 and AMPK can be seen in the case of the liver, in which loss of LKB1 causes marked hyperglycemia, whereas mice with liver-specific loss of both AMPK catalytic subunits are normoglycemic. The more severe phenotype caused by ablation of LKB1 rather than AMPK in the liver may be due to reduced activity of SIK1, which as mentioned earlier regulates expression of gluconeogenic genes [46].

## The role of AMPK in cancer

As pointed out in an accompanying mini-review at the time [47], perhaps the biggest impact of our paper and the others linking LKB1 and AMPK [1-3] came from the link they established between a tumor suppressor (LKB1) with roles in cancer, and a protein kinase (AMPK) that had previously been regarded as a regulator of metabolism, with clinical implications in diabetes but not in cancer. While such a link had been suggested previously by pioneering work from the Esumi laboratory [48,49], the three simultaneous reports that LKB1 acted upstream of AMPK brought it into sharper focus. A key question was whether the tumor suppressor functions of LKB1 were mediated by AMPK, or by one or more of the ARKs, or both. This has still not been completely resolved, although there are good reasons for expecting that AMPK may mediate some of the tumor suppressor functions of LKB1. Indeed, a tumor suppressor function for AMPK is supported by a recent paper reporting that a whole-body knockout of AMPK-α1, which is the only catalytic subunit expressed in B cells, accelerates the development of lymphomas in transgenic mice overexpressing c-Myc in the B cells [50]. More generally, actions of AMPK that suggest it may be responsible for some of the tumor suppressor functions of LKB1 are as follows.

First, AMPK activation causes a cell cycle arrest associated with stabilization of p53 and the cyclin-dependent kinase inhibitors p21^WAF1^ and p27^CIP1 ^[49,51,52]. Second, AMPK activation inhibits the synthesis of most cellular macromolecules, including fatty acids, triglycerides, cholesterol, glycogen, ribosomal RNA and proteins [53,54], thus inhibiting cell growth. It is particularly significant in this respect that AMPK inhibits the mechanistic target-of-rapamycin complex-1 (mTORC1) by phosphorylating its upstream regulator TSC2 [55] and its regulatory subunit Raptor [56] and thus inhibits translation of many proteins required for rapid cell growth, including hypoxia-inducible factor-1α (HIF-1α). This latter effect contributes to the third potential tumor suppressor action of the enzyme - the 'anti-Warburg' effect. The Warburg effect is the switch away from oxidative metabolism and towards rapid glucose uptake, glycolysis and lactate output that is characteristic of most tumor cells [5]. Although AMPK activation can acutely increase glucose uptake [57] and glycolysis in certain cells [58,59], in the longer term it promotes the more energy-efficient oxidative metabolism, by up-regulating mitochondrial biogenesis [60] and expression of oxidative enzymes [61], while down-regulating the glycolytic pathway by inhibiting mTORC1. Inhibition of mTORC1 decreases translation of HIF-1α [62], a transcription factor that drives expression of enzymes and transporters required for the Warburg effect, including most glycolytic enzymes as well as the transporters GLUT1 and MCT4, which are required for glucose uptake and lactate output, respectively. Consistent with the idea that AMPK exerts an 'anti-Warburg' effect, expression of HIF-1α and downstream glycolytic genes are up-regulated in LKB1-null or AMPK-α1 -α2 double null mouse embryo fibroblasts [63]. The Warburg effect is also enhanced in the lymphoma cells mentioned earlier, derived by c-Myc over-expression in B cells when AMPK was knocked out *in vivo*, as well as in lung and colon cancer cells in culture when AMPK is knocked down *in vitro *[50].

If the LKB1-AMPK pathway does indeed act as a tumor suppressor that normally restrains growth and proliferation of cancer cells as well as the associated metabolic changes, one would expect such cells to be under selective pressure to down-regulate the pathway. One obvious mechanism by which this happens is genetic loss of LKB1 due to somatic mutations which, as shown in our 2003 paper [1], leads to failure of AMPK activation following metabolic stresses that increase AMP and ADP (loss of LKB1 would, of course, down-regulate the ARKs as well as AMPK). Such mutations are now estimated to occur in approximately 30% of non-small cell lung cancers [64,65], approximately 20% of cervical cancers [66], and approximately 10% of cutaneous melanomas [67]. There are also other mechanisms by which the pathway can be down-regulated in cancer, through direct effects on AMPK rather than as downstream consequences of LBK1 inactivation. Thus, expression of the AMPK-α2 subunits is reduced in some cases of hepatocellular carcinoma, and this is associated with enhanced tumor cell growth in mouse xenografts, and poorer patient prognosis [68], while in melanoma cells that carry the V600E mutation in B-Raf, LKB1 appears to be phosphorylated at carboxy-terminal sites, and this is associated with reduced AMPK activation [69].

A further potential mechanism of AMPK down-regulation that may contribute to tumorigenesis depends on an inhibitory effect of phosphorylation at Ser485 by Akt, which has been reported to prevent the activating phosphorylation at Thr172 [70]. This might occur in tumors in which Akt is hyper-activated due to loss-of-function mutations in the lipid phosphatase PTEN, or activating mutations in phosphoinositide-3-kinase (PI3K) [71]. Although this mechanism has not yet been studied in the context of cancer, it has been shown to operate in human hepatoma cells infected with hepatitis C virus (HCV), in which Akt is activated due to interactions between PI3K and one of the virus-encoded non-structural proteins, NS5A [72]. This causes a marked phosphorylation of Ser485 on AMPK-α1 and reduced Thr172 phosphorylation. Intriguingly, expression of an S485A AMPK-α1 mutant in the cells reduces expression of viral protein, suggesting that phosphorylation of AMPK at Ser485 is required for efficient viral replication [73]. Up to 80% of individuals infected with HCV develop a chronic infection, which greatly increases their risk of fatty liver disease and hepatocellular carcinoma [72]. Since activation of fatty acid oxidation and inhibition of fatty acid synthesis are two of the classical effects of AMPK [11], while AMPK can also act as a tumor suppressor, down-regulation of AMPK by HCV may help to explain the increased risk of both fatty liver disease and liver cancer.

## AMPK, metformin, and the prevention or treatment of cancer

**Figure 5:**
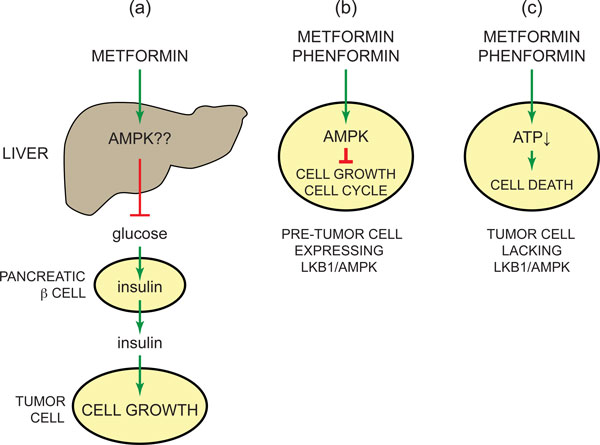
**Three possible mechanisms to explain how the AMPK-activating drugs metformin or phenformin might provide protection against cancer**. **(a) **Metformin acts on the liver and other insulin target tissues by activating AMPK (and probably via other targets), normalizing blood glucose; this reduces insulin secretion from pancreatic β cells, reducing the growth-promoting effects of insulin (and high glucose) on tumor cells. Since metformin does not reduce glucose levels in normoglycemic individuals, this mechanism would only operate in insulin-resistant subjects. (**b) **Metformin or phenformin activates AMPK in pre-neoplastic cells, restraining their growth and proliferation and thus delaying the onset of tumorigenesis; this mechanism would only operate in cells where the LKB1-AMPK pathway was intact. (**c) **Metformin or phenformin inhibits mitochondrial ATP synthesis in tumor cells, promoting cell death. If the LKB1-AMPK pathway was down-regulated in the tumor cells, they would be more sensitive to cell death induced by the biguanides than surrounding normal cells.

If the LKB1-AMPK pathway is indeed a tumor-suppressing pathway, then AMPK-activating drugs might be expected to provide protection against the development of cancer. Indeed, our 2003 paper [1], taken together with the earlier observations that metformin activates AMPK [13], led directly to an investigation in which it was found that type 2 diabetics treated with the AMPK-activating drug metformin had a significantly reduced incidence of all forms of cancer [74]. This has been reproduced in several subsequent studies of different diabetic populations, with a meta-analysis indicating an overall summary risk reduction of 30%, with specific risk reductions being found for colon and liver cancers [75]. It should be noted that these studies merely report associations between cancer incidence in diabetics treated with metformin compared with those on other medications (typically sulphonylureas or insulin), and do not prove a causal link. They have also been criticized on the basis that they may be subject to time-related biases [76]. However, there are, as we have seen, other reasons for supposing that activation of AMPK may suppress tumorigenesis, and in this light there are at least three mechanisms that might explain its protective effects (Figure 5). The first mechanism (Figure 5a) involves the indirect action of metformin, through effects at sites other than the tumors themselves (especially the liver), while the other two (Figure 5b,c) involve direct effects of metformin on the pre-tumor or tumor cells. It is worth pointing out that these mechanisms are not mutually exclusive and all could contribute to an overall effect, although the second two mechanisms could not co-exist in a single tumor cell.

The first of the possible mechanisms (Figure 5a) derives from the observation that cancer incidence is increased in type 2 diabetics, and the contribution of AMPK in this case is not yet clear. In type 2 diabetes, reduced insulin sensitivity of insulin target tissues causes a compensatory increase in the secretion of insulin. Insulin is of course a growth factor, and there is a positive association between plasma levels of insulin and cancer [77], suggesting that elevated insulin (and perhaps also glucose) could be the cause of increased cancer in type 2 diabetics. In this case, metformin could reduce cancer incidence by enhancing the insulin sensitivity of insulin target tissues, causing normalization of blood glucose and reducing hyper-secretion of insulin from pancreatic β cells. By contrast, other treatments, including sulphonylureas (which enhance insulin release from β cells), and injection of insulin, will tend to increase plasma insulin. Some support for this mechanism comes from studying growth of colon carcinoma (MC38) cells as mouse xenografts. When the mice are fed a high-fat diet to render them insulin-resistant and thus increase plasma insulin, growth of the implanted tumors is enhanced; metformin treatment reverses both the increase in plasma insulin and the tumor growth rate [78]. In such xenograft studies, the tumor cells of course already exist, and insulin is merely affecting their growth rate.

## Direct effects of biguanides in tumor cells and the emergence of a paradox

Other studies suggest that metformin might delay the initial development of tumors by direct actions on the tumor cells themselves, even in animals that are not insulin-resistant. For example, in mice that are tumor-prone due to heterozygous loss of PTEN combined with reduced expression of LKB1, development of tumors (mostly lymphomas) can be delayed by treating the mice from the time of weaning with metformin or phenformin, or by another AMPK activator, A-769662 [79]. Since the biguanides and A-769662 activate AMPK by different mechanisms [14], it is unlikely that the delay in tumorigenesis is due to off-target, AMPK-independent effects. In addition, neither class of drug activates any of the ARKs, making it unlikely that their protective effects have anything to do with other kinases downstream of LKB1. One possible mechanism for this effect (Figure 5b) is that activation of AMPK exerts a cytostatic effect on pre-neoplastic lesions through its ability to inhibit cell growth and progress through the cell cycle, and in this way delays the onset of tumorigenesis.

An alternative mechanism whereby biguanides could lead to the suppression of tumor growth derives from the inhibition by these drugs of the mitochondrial respiratory chain (Figure 5c) [14,80], although this action depends, paradoxically, not on the activation of the LKB1-AMPK pathway but on its down-regulation. Because cells with a defective LKB1-AMPK pathway are less able to restore ATP levels in response to metabolic stress [44,81,82], tumor cells in which the pathway has been down-regulated may be less able to adapt to mitochondrial inhibition by biguanides, and thus more susceptible to cell death. There is some direct experimental support for this mechanism in the mouse xenograft study of MC38 colon carcinoma cells mentioned earlier [78], in which treatment with metformin reduced the rate of tumor growth in insulin-resistant mice: growth of the xenografts was also slowed by metformin even when the mice were not insulin-resistant, but only if LKB1 had been first knocked down in the tumor cells using RNA interference. In another elegant study involving a mouse model of non-small cell lung cancer, phenformin prolonged survival of the mice when tumors were induced by activation of mutant K-Ras combined with loss of LKB1, but not when the mutant K-Ras was combined with loss of p53 [83]. Phenformin was used in this study because it is a more potent inhibitor of the respiratory chain than metformin [80], and appears to be less dependent on the expression of specific membrane transporters for its cellular uptake [14]. This again is consistent with the proposal that biguanides kill LKB1-deficient tumor cells because they are more sensitive to their ATP-depleting effects than the surrounding normal cells, which would still have a functional AMPK pathway. (Interestingly, cells classed as 'cancer stem cells' appear to be particularly sensitive to metformin, although the molecular mechanism underlying this is unclear [84,85].)

## AMPK - tumor suppressor or tumor promoter?

Since our paper and the others [1-3] published nearly ten years ago suggested a link between AMPK and cancer, the once separate worlds of AMPK and metabolism, and mitogenic signaling in cancer cells have undergone a remarkable fusion. Considerable excitement has also been generated by the realization that inexpensive biguanide drugs with manageable side effects might have new applications in cancer. Pilot 'window-of-opportunity' trials of metformin in breast cancer have already been conducted [86,87], and much more ambitious prospective trials are planned.

However, it is also becoming apparent, as seen in the papers described above, that under different circumstances AMPK can be either a suppressor or a promoter of cancer. In the light of recent evidence, it seems possible that this may depend upon the stage in the development of the tumor. In pre-neoplastic lesions AMPK may act as a tumor suppressor, limiting cell growth and the switch from oxidative metabolism to the Warburg effect, and thus preventing conversion to full-blown tumors: in this case, the pre-neoplastic cells will be under strong selective pressure to down-regulate the LKB1-AMPK pathway, explaining the observed inactivation of LKB1 in a significant proportion of tumors. Paradoxically, however, if a tumor becomes established without losing the function of the LKB1-AMPK pathway, AMPK may help to keep the tumor cells alive by protecting them from metabolic stress. There is recent evidence in favor of the latter proposition. In a study of LKB1-deficient lung adenocarcinoma (A549) cells, it was found that re-expression of LKB1 protected the cells against cell death induced by glucose starvation, apparently through inhibition of fatty acid synthesis by AMPK and consequent sparing of NADPH, which could be utilized to provide protection against the oxidative stress induced by glucose deprivation [88]. In another study [89], both AMPK and the AMPK-related kinase NUAK1 (Ark5) were detected in a synthetic lethal RNA interference screen for kinases whose knock-down caused apoptosis of osteosarcoma (U2OS) cells over-expressing c-Myc. This suggests that AMPK and NUAK1 normally help to protect these cells against the stresses caused by the metabolic reprogramming that accompanies c-Myc over-expression.

Thus, the LKB1-AMPK pathway may act as a tumor suppressor through its ability to restrain the growth of pre-neoplastic lesions, but paradoxically may make established tumor cells more resistant to the metabolic stresses induced by biguanides or cytotoxic drugs, or that occurs when growth of the tumor outstrips the capacity of its blood supply to deliver oxygen and nutrients. It thus seems possible that the use of AMPK activators might be harmful in such cases, and there might be a need instead for specific inhibitors of AMPK.

This view is consistent with the recent study of a mouse model of non-small cell lung cancer already discussed above [83], which suggested that biguanides might be an effective method to treat tumors in which the function of the LKB1-AMPK pathway has been lost. In this situation, biguanides are acting not as AMPK activators but as cytotoxic drugs that deplete cellular ATP. This study also suggested that phenformin might be more effective than metformin in this regard, because it is more cell-permeable and a more potent inhibitor of the respiratory chain. Although phenformin was withdrawn for treatment of diabetes due to cases of lactic acidosis, this side effect was rare (<1 case per 1,000 patient-years) and might be more acceptable in treatment of cancer. Overall, a better understanding of the way in which the varied actions of AMPK interact with the different metabolic requirements of cancer cells is clearly essential to allow the more rational design of clinical trials of metformin and other AMPK-modulating drugs.

## Future directions, both upstream of AMPK and downstream of LKB1

The other major findings that emerged in the immediate aftermath of our 2003 paper were that LKB1 also acted upstream of the AMPK-related kinases or ARKs, and that AMPK could also be activated by the Ca^2+^-CaMKK pathway. Although the functions of the ARKs have been slowly emerging since then, their regulation remains poorly understood. LKB1 appears to be constitutively active, and in the case of AMPK the regulation of Thr172 phosphorylation is brought about by binding of ligands to AMPK, and not to LKB1. In the case of the ARKs, we do not understand how their phosphorylation by LKB1, and hence their activity, is regulated. Much also remains to be learned about the physiological role of AMPK activation by the Ca^2+^-CaMKK pathway. One intriguing question is whether inhibition of cell growth and proliferation by AMPK can be triggered by this pathway in tumor cells that have lost LKB1.

## Note

This article is part of the *BMC Biology *tenth anniversary series. Other articles in this series can be found at http://www.biomedcentral.com/bmcbiol/series/tenthanniversary.
